# Supplementary material for: Chromothripsis during telomere crisis is independent of NHEJ, and consistent with a replicative origin
Source: Genome Res. 2019 May;29(5):737–49. doi: 10.1101/gr.240705.118 (PMC6499312; doi:10.1101/gr.240705.118)
Supplement: Supplemental Material [file supp_gr.240705.118_Supplemental_file_1.zip › contigs/annotated_contigs/DB69/contig.4.DB69_length_842_mean_cov_22.5023752969.docx]

**DB69_length_842_mean_cov_22.5023752969**

```
CACATGTATGTTTATTGCAGCACTATTCACAATAGCAAAGACTTGGAACCAACCCAAATGTCCATCAATGATAGAACTGATTAAGAAAA
 >chr9:66478618-66478734 - E=7e-55 p=0e+00
TGCAGCACGTATACACCATGGAATAG|T|GTAGAGACAGGGTTTCACCGTGTTAGCCAGGATGGTCTCGATCTCCTGACCCTGTGATCC
                           >chr9:66469907-66470439 + E=3e-308
CCCCACCCTGGCCTCCCAAAGTGCTGGGATTACAGGCGTGAGCCACTGTGCCTGGCTCGTCTTCTTTTGAGAAGTGTCTGCTTATGTCC

CTTGTCAATGTTTTAATGGGGTTGTTTTCCTAATGTCCAACATCACTAATCATCAGAGAAATGCAAGTCAATGAGAGATACCATCTCAT

ATTAGTCAGAATGGCTACTATTAAAAAGTAAAAAAATGACAAATTCTGGTTAGACTTCAGAGAAAATGAAATGCTTATACACTGTTGGT

GAGAATGTAAATTAGTTCAGCCACTGTGAAAAGCAGTTTGGAGATTTTTCAAAGAATTTAAAACAGAAGTGCCATTCAACCAGCAATTC

CATTACTGAGTATATATCCAAAAGAAAATTAAGAATTCCACAAAAAAGAAACCCCACATGCACTTGTATATGAGTCATATTCTATTCAC

AATAGGGAAGGCATGGAATTAAC|TTA|ATGTGTGCATAGTGTTTTATGGCGTATATGTGGCACATATTTCTATC|CA|CACACACACA
                        >chr9:66470456-66470508 - E=2e-19                 >chr8:85337
CACACACACACACACACACACATAGAAGAAAAACTGGCTTTCTAAAAATTGATGCTCCAATTGCATGCTATTGTTGGATTGATCATGTT
906-85338054 - E=4e-71
TCTGGAGGGTCTCTGGAGTGACAATCTTCCAATAGCATCAGGATAGG
```
